# Autologous Stem Cell Transplantation for Myeloma: Cytoreduction or an Immunotherapy?

**DOI:** 10.3389/fimmu.2021.651288

**Published:** 2021-03-12

**Authors:** Simone A. Minnie, Geoffrey R. Hill

**Affiliations:** ^1^Clinical Research Division, Fred Hutchinson Cancer Research Center, Seattle, WA, United States; ^2^Division of Medical Oncology, University of Washington, Seattle, WA, United States

**Keywords:** myeloma, stem cell transplantation, immunotherapy, T cells, autologous

## Abstract

The incidence of multiple myeloma (MM), a bone marrow (BM) resident hematological malignancy, is increasing globally. The disease has substantial morbidity and mortality and remains largely incurable. Clinical studies show that autologous stem cell transplantation (ASCT) remains efficacious in eligible patients, providing a progression free survival (PFS) benefit beyond novel therapies alone. Conventionally, improved PFS after ASCT is attributed to cytoreduction from myeloablative chemotherapy. However, ASCT results in immune effects beyond cytoreduction, including inflammation, lymphodepletion, T cell priming *via* immunogenic cell death, and disruption of the tumor BM microenvironment. In fact, a small subset of patients achieve very long-term control of disease post-ASCT, akin to that seen in the context of immune-mediated graft-vs.-myeloma effects after allogeneic SCT. These clinical observations coupled with recent definitive studies in mice demonstrating that progression after ASCT represents immune escape as a consequence of T cell exhaustion, highlight the potential for new immunotherapy maintenance strategies to prevent myeloma progression following consolidation with ASCT.

## Introduction

Autologous stem cell transplantation (ASCT) occurs after treatment with varying combinations of proteasome inhibitors, alkylating agents, immunomodulatory drugs (IMiDs), steroids and most recently, monoclonal antibodies until a maximal response is achieved. At that point, eligible patients typically undergo G-CSF-based stem cell mobilization followed by autologous stem cell collection and storage. Patients then receive myeloablative chemotherapy, predominantly high-dose melphalan (1), followed by autologous stem cell rescue and subsequent maintenance therapy with an IMID, typically lenalidomide. This regimen remains a highly effective therapy and, despite recent advances in anti-myeloma therapeutics, ASCT provides a progression-free survival benefit beyond novel agents alone (2–5). Hitherto, the control of myeloma progression induced by ASCT is largely attributed to the direct cytoreductive effects of myeloablative chemotherapy on myeloma cells (6). However, there is a subset of patients that enter a plateau-phase of disease control after achieving a complete response to ASCT, akin to immune-mediated graft-vs.-leukemia effects after allogeneic-SCT (7, 8). In fact, there are several key immunological changes that occur after ASCT that strongly suggest that long-term myeloma control after transplant is due to more than just cytoreduction. In this perspective, we will outline key evidence from both clinical observations and definitive preclinical studies that support the concept that ASCT sets the stage for myeloma-specific immunity.

## Immunological Consequences of ASCT

### Immune Reconstitution and Myeloma-Specific T Cells

Natural killer (NK) cells usually reconstitute to normal levels within 1 month of ASCT; much faster than adaptive immune cells such as B and T cells (9, 10). Importantly, NK cell dysfunction has been associated with myeloma progression in non-transplant murine models and patients with late-stage myeloma have reduced NK cell numbers suggesting that these cells may play an important role in myeloma control (11–13). NK cells have been shown to be especially important in the context of IMiDs such as thalidomide, lenalidomide, and pomalidomide, since these agents stimulate IL-2 production that promotes NK cell expansion and activation (14–16). However, NK cell-mediated myeloma immunity was found to be dispensable for myeloma control after ASCT in a murine model when donor T cells were transplanted (17). Therefore, although NK cells are potent mediators of myeloma immunity, they may not be critical when a robust T cell response is generated post-ASCT. Whether this holds true in patients after ASCT, particularly in those receiving maintenance therapies with IMIDs requires further investigation.

T cell reconstitution occurs more slowly after ASCT and can take up to a year to return to pre-transplant numbers (10). Interestingly, the recovery of T cell subsets occurs more rapidly after transplantation with peripheral blood stem cells (PBSC) compared to bone marrow (BM) (9, 10). In fact, CD3^+^ T cells represent >20% of the apheresis product collected after granulocyte colony-stimulating factor (G-CSF) mobilization and subsequently PBSC grafts yield substantially (>1 log) more T cells than BM grafts (18, 19). More importantly, the T cell reconstitution after ASCT is skewed toward CD8 T cells and provides a favorable CD8 effector T cell (Teff) to Treg ratio (20, 21) which often underpins effective anti-tumor immunity. Indeed, the addition of T cells to BM grafts dramatically improved survival and reduced myeloma burdens in a murine model of ASCT (17). While this effect was dependent on both CD8 and CD4 T cells, CD8 T cells were the cognate effectors of myeloma-specific immunity (17). Additionally, pre-existing memory T cells from myeloma-experienced donor mice were the dominant mediators of myeloma-specific immunity after ASCT; although myeloma-specific T cells could also be generated *de novo* after ASCT from naïve T cells present in the graft (17). Clinical studies also support a role for memory and effector T cells in mediating myeloma-specific immunity, particularly in the context of progression from MGUS to MM (22, 23). Furthermore, there are detectable myeloma-reactive T cells in patients with myeloma after ASCT and the expansion of cytotoxic T cell clones after treatment with IMiDs has been associated with improved outcomes (24–26). Together these data suggest that T cells contaminating stem cell grafts could be the predominant mediators of myeloma-specific immune responses post-transplant. This carries significant potential clinical impact as induction therapies are currently implemented without regard for T cell recovery or function in the subsequently mobilized donor stem cell graft.

### Microenvironment Disruption and Changes in Cytokine Production

Progression of malignancy is typically associated with the development of an immunosuppressive tumor microenvironment (TME) capable of subverting effective anti-tumor immunity (27). Myeloma also generates an immunosuppressive BM environment that is reminiscent of that seen in solid tumors (28–30). As an example, the accumulation of BM macrophages has been shown to protect myeloma from apoptosis (31). Melphalan is most commonly used in conditioning before ASCT due to its potent anti-myeloma cytotoxicity (32). The minimal residual disease state, concurrent with disruption of the bone marrow microenvironment following ASCT thus provides a potential window of opportunity to generate effective myeloma-specific immunity (33), mediated by T cells (17).

Myeloablative conditioning preceding ASCT transiently depletes regulatory T cells which has been shown to improve the anti-tumor efficacy of adoptively transferred CD8 T cells in preclinical models (34, 35). However, conditioning itself also results in a profound state of lymphodepletion which itself has important ramifications for the generation of myeloma-specific immunity. In particular, extensive cytoreduction minimizes endogenous cellular competition for cytokine which results in high cytokine availability to newly transferred T cells, enhancing both proliferation and effector function (i.e., homeostatic proliferation) (35). Specifically, this effect is mediated by increased availability of both IL-7 and IL-15 since the ablation of both cytokines abrogated enhanced anti-tumor efficacy seen in irradiated vs. untreated mice. In patients with myeloma, it has been shown that high-dose melphalan and ASCT results in increased plasma levels of IL-6, IL-7, and IL-15. Furthermore, and in support of preclinical findings, Condomines et al. postulated that this increase in IL-7 and IL-15 may contribute to the activation and survival of transplanted T cells from the donor graft (36). In mice, melphalan also increases plasma levels of several additional cytokines including IFN-γ, IL-27, IL-5, IL-22, IL-10, and IL-18, as well as chemokines such as CCL2, CCL7, CXCL10, and CXCL1; which augments CD4 T cell-dependent immunity (37). Thus, cytoreduction and lymphopenia after ASCT are associated with changes in cytokine production and immune activation which contribute to subsequent tumor-specific immunity.

### Immunogenic Cell Death and Antigen Presentation

Immunogenic cell death (ICD) is classically characterized by surface expression of calreticulin (CRT), release of ATP, and secretion of high mobility group box 1 (HGMB1) from the nucleus (38). This stimulates DC recruitment to the TME that enhances phagocytosis and subsequent (tumor-derived) antigen presentation (38, 39). The induction of ICD by cyclophosphamide and bortezomib, two drugs commonly used in myeloma, is well-described however it is less clear whether melphalan has similar effects (40, 41). In myeloma, melphalan has been shown to induce exosome release and cytokine production from NK cells in an HSP70-dependent manner (42). A preclinical study in mice found that melphalan induced ICD, which was associated with the release of inflammatory cytokines and enhanced antigen uptake by DCs (37). Specifically, CRT was expressed on the surface of a B-cell lymphoma cell line in response to melphalan with extracellular release of HMGB1. Melphalan also resulted in activation of CD8 T cells due to increased antigen uptake and presentation by tumor associated DCs, Treg depletion and a transient reduction in myeloid-derived suppressor cells (MDSC) (37). This provides preclinical evidence that melphalan induces desirable immunomodulatory activities akin to other alkylating agents like cyclophosphamide. However, it should be noted that melphalan may not only elicit beneficial immunomodulatory effects. For example, melphalan-induced IL-6 production could be problematic given it behaves as a growth factor for myeloma and may contribute to immune escape post-ASCT (17).

## Immune Escape in the Context of ASCT

We postulate that ASCT establishes a state of myeloma-immune equilibrium followed by immunological escape and subsequent myeloma progression. Immunological escape is known to be facilitated by a multitude of factors including alterations in cytokine production, T cell exhaustion, and accumulation of macrophages and MDSCs within the TME (43–45). Immune escape in the broad context of myeloma has been reviewed elsewhere (46, 47) and this perspective will focus on immune escape that may occur specifically in the context of ASCT.

### Interleukin-6

T cell-mediated myeloma control can be influenced by alterations in the cytokine milieu in the TME that either directly suppress effector T cell function or *via* alteration of differentiation such that cytolytic T cell subsets are replaced by those that promote tumor growth. One such cytokine is IL-6, which is known to not only directly promote myeloma growth and survival, but also contributes to the expansion of pathogenic T helper 17 (Th17) cells (48–50). Additionally, IL-6 is known to confer resistance in myeloma cells to bortezomib, melphalan, and in particular, dexamethasone-induced apoptosis (51–53). Interestingly, there is both clinical and preclinical evidence that IL-6 is one of the cytokines markedly upregulated by melphalan (36, 37, 54). Given the myeloma-promoting properties of IL-6, disrupting this pathway appeared to be an attractive approach to improve myeloma control. In mice, IL-6 deficient BM and T cell grafts significantly reduced myeloma relapse post-ASCT (17). However, in patients with relapsed-refractory multiple myeloma (RRMM), there was no benefit to the addition of siltuximab, an IL-6 inhibitor, to bortezomib and/or dexamethasone-containing treatment regimes in non-transplant settings (55, 56). A study utilizing preclinical myeloma models demonstrated that siltuximab enhanced melphalan-mediated cytotoxicity (52); possibly by mitigating melphalan-induced IL-6 production. Nonetheless, although IL-6 inhibition is capable of impacting myeloma growth *in vitro* and in preclinical models, it has been largely unsuccessful in the clinical setting; which may be unsurprising given the immune-suppressive nature of an established TME in RRMM. This strategy will likely continue to be ineffective without also promoting a state of minimal residual disease (MRD) and immune-mediated clearance of tumor, the latter being particularly challenging in a relapsed/refractory setting due to the concurrence of T cell exhaustion. Thus, studies combining IL-6 inhibition with immunotherapies such as ASCT, immune checkpoint blockade or CAR T cells could be potentially synergistic and may warrant further investigation.

### Microbiota

There is increasing clinical evidence for the role of the microbiome in dictating responses to immunotherapy and even endogenous tumor immunity (57). This is of particular relevance in the context of transplantation where microbial diversity in the GI tract is dramatically impacted by conditioning regimens and antibiotic exposure (58, 59). Patients undergoing ASCT for myeloma, lymphoma, or amyloidosis who had above-median microbial diversity in the GI tract had a reduced risk of progression suggesting a relationship between microbiota and patient outcomes (58). Preclinical data further supports a role for the microbiome in myeloma progression whereby migration of pathogenic IL-17-producing cells to the BM is driven by *Prevotella heparinolytica* to promote myeloma growth (60). We demonstrated that IL-17A, a highly microbiota-dependent cytokine (61), is pathogenic after ASCT and acts directly on myeloma cells in the BM to promote relapse (17). In mice, IL-17A inhibition with monoclonal antibodies (mAbs), both in untreated myeloma and after ASCT, attenuates myeloma progression (17, 60), thus representing a potential therapeutic strategy that is under clinical investigation (NCT03111992).

### T Cell Exhaustion

There is significant preclinical and clinical evidence to support a role for CD8 T cell exhaustion/dysfunction, including loss of IFNγ and CD107a production, in facilitating myeloma progression (21, 29, 62–64). Additionally, there are several studies that have found CD8 T cells with increased expression of immune checkpoint receptors, including TIGIT, PD-1, LAG-3, TIM-3, and CTLA-4 in patients with myeloma (21, 62, 63, 65, 66). In the context of ASCT, one study found that inhibitory receptors are expressed both pre- and post-ASCT with increased PD-1 on a subset of CD8 T cells after ASCT (21), while another reported an increase in LAG-3 expression post-ASCT (67). Furthermore, there is clinical evidence that ASCT induces global transcriptional changes in peripheral CD4 and CD8 T cells that are associated with a reduced CD4/CD8 ratio and enhanced T helper 1 differentiation, exhaustion, activation, senescence, and molecular aging (67–69). The functional implications of these latter phenotypes, particularly increased molecular aging after ASCT are currently unclear.

Preclinical data suggests that ASCT both enhances pre-existing myeloma-specific T cell immunity derived from the stem cell graft and primes new myeloma-specific T cells after transplant. Myeloma progression after SCT is associated with T cell exhaustion, such that the prevention of T cell exhaustion in the peri-transplant window is crucial to promote long-term disease control. This is supported by several murine models of myeloma that demonstrate some immunotherapies are ineffective unless utilized in combination in the setting of RRMM. Alternatively, demonstration of single agent efficacy requires administration early after ASCT [i.e., anti-PD-1 (29, 62)] with a loss of efficacy seen when initiated when significant myeloma burdens exist (17, 70). The lack of efficacy of PD-1 blockade in patients with active myeloma, either as a monotherapy or in combination with IMiDs further support this concept (71, 72). Thus, there is a critical need to further study CD8 and CD4 T cell function, phenotype, and myeloma-specific immunity within the BM TME of myeloma patients after ASCT in order to define the optimal timing for immunotherapy approaches. Notably, new immunotherapy approaches will need to be administered in conjunction with existing standard-of-care maintenance therapies (e.g., lenalidomide). Previous combinations of pembrolizumab and lenalidomide were associated with toxicity in patients with relapsed/refractory myeloma (72) and so new combinations and the timing of their administration with the disease course (e.g., early vs. late) will need to be carefully considered. Since some immune checkpoint inhibitors are likely to be more effective than others when combined with IMiDs, due to non-overlapping immunological mechanisms of action and toxicities, there is a need for comprehensive preclinical testing to provide a clear rationale for potential clinical combinations.

### Suppressive Myeloid Populations

Cytoreductive therapy can invoke undesirable immunological effects, including the accumulation of suppressive myeloid populations within the TME; an effect described in both preclinical models and patients (73–75). In myeloma, colony stimulating factor 1 receptor (CSF-1R) expressing tumor associated macrophages (TAM) have been shown to promote disease progression and CSF-1R inhibition prolongs myeloma-specific immunity, particularly after ASCT (29, 31, 76). Myeloid-derived-suppressor cells (MDSC) are also key inhibitors of anti-tumor immune responses and are increased in the bone marrow of patients with myeloma where they suppress T-cell-mediated immune responses (77, 78). Studies also suggest that there is a bi-directional relationship between MDSC and myeloma whereby the myeloma cells induce differentiation of healthy PBMCs into MDSC (78). Importantly, a study utilizing S100A9 knockout mice, which do not accumulate MDSC in the TME, found that early MDSC accumulation in the BM was sufficient to inhibit myeloma antigen-specific CD8 T cell responses (77). Clinically, the role of circulating MDSCs on ASCT outcomes has recently been described in a cohort of 100 patients (79). The study showed that increased monocytic MDSCs (M-MDSCs) prior to ASCT were associated with a shorter time to progression post-transplant. Further *in vitro* studies, where myeloma cells were co-cultured with M-MDSCs and then treated with melphalan, suggested that this was associated with the ability of M-MDSCs to protect myeloma cells from melphalan-mediated cytotoxicity. This M-MDSC-mediated protection was mitigated by CSF-1R blockade *in vitro*, although this effect was not confirmed *in vivo* (79). Together, these data support the pathogenicity of CSF-1R-expressing macrophages and MDSCs in myeloma, particularly in the context of ASCT, and highlight a clinically tractable population to improve the depth and duration of immune responses after transplant.

Suppressive myeloid cells are also known to express CD38, the target for several FDA approved monoclonal antibodies including daratumumab and isatuximab. CD38 is also expressed on myeloma cells and regulatory T cells; the depletion of all three cellular compartments is thought to underpin the promising clinical efficacy of this class of drugs (80–83). Response rates to CD38 mAbs are consistently encouraging in myeloma when combined with current standard of care (84, 85), even in the relapsed/refractory setting (80, 81), such that the addition of daratumumab to standard induction therapy (pre-ASCT), and consolidation post-ASCT has recently been reported (86, 87). Early results from two clinical trials (CASSIOPEIA and GRIFFIN) studying the addition of daratumumab in this fashion have shown an increase in the frequency of MRD negative responses, and a larger phase 3 randomized trial is currently underway (NCT03710603) (86, 87). However, these studies lack long-term follow up and the broader immunological consequences of depleting putatively activated, myeloma-specific CD38-expressing CD8 T cells has yet to be ascertained (88). Given the striking efficacy of CD38 mAbs in combination with current therapies, the loss of CD38^+^ CD8 T cells may be manageable. Nevertheless, in circumstances where the activation of T cell-specific immunity is being actively elicited for therapeutic benefit (e.g., immune checkpoint inhibition), combination therapy will require more careful consideration.

## Conclusion: ASCT is a Platform Immunotherapy

High-dose chemotherapy, particularly melphalan, produces immunomodulatory effects including inflammatory cytokine production, immunogenic myeloma cell death, enhanced antigen presentation, microenvironment disruption and lymphodepletion. Together, these effects culminate in an ideal environment for subsequent priming, activation and expansion of transplanted donor T cells. The combined immunological and cytoreductive consequences of ASCT as consolidation therapy for myeloma thus positions this therapy as an ideal platform for maintenance immunotherapy with the aim of amplifying immune control and preventing or delaying disease progression. Thus, T cell targeted immunotherapies such as immune checkpoint inhibitors, monoclonal antibodies, tumor vaccination, bispecific T cell engagers and CAR T cells [reviewed elsewhere (46)] are all viable strategies to prevent relapse and could be trialed as consolidation and/or maintenance therapies after ASCT, once early phase safety data has been established. Importantly, restricting the use of novel agents that rely on a competent T cell response, or at the very least reversible T cell exhaustion, to relapsed/refractory patients where irreversible stage T cell dysfunction invariably exists is likely to limit the development of agents that may be highly effective in earlier settings of minimal residual disease. Finally, these principals also suggest that the ability of various induction therapies (and combinations thereof) to preserve immune competence prior to and during stem cell mobilization deserves further consideration.

## Author Contributions

SM and GH wrote and edited the manuscript.

## Conflict of Interest

GH has received research funding from Compass Therapeutics, Roche, iTeos and Syndax. The remaining author declares that the research was conducted in the absence of any commercial or financial relationships that could be construed as a potential conflict of interest.
